# High‐throughput methods for efficiently building massive phylogenies from natural history collections

**DOI:** 10.1002/aps3.11410

**Published:** 2021-02-27

**Authors:** Ryan A. Folk, Heather R. Kates, Raphael LaFrance, Douglas E. Soltis, Pamela S. Soltis, Robert P. Guralnick

**Affiliations:** ^1^ Department of Biological Sciences Mississippi State University Mississippi State Mississippi USA; ^2^ Florida Museum of Natural History University of Florida Gainesville Florida USA; ^3^ Department of Biology University of Florida Gainesville Florida USA; ^4^ Genetics Institute University of Florida Gainesville Florida USA; ^5^ Biodiversity Institute University of Florida Gainesville Florida USA

**Keywords:** destructive sampling, herbaria, herbariomics, museomics, phylogenomics

## Abstract

**Premise:**

Large phylogenetic data sets have often been restricted to small numbers of loci from GenBank, and a vetted sampling‐to‐sequencing phylogenomic protocol scaling to thousands of species is not yet available. Here, we report a high‐throughput collections‐based approach that empowers researchers to explore more branches of the tree of life with numerous loci.

**Methods:**

We developed an integrated Specimen‐to‐Laboratory Information Management System (SLIMS), connecting sampling and wet lab efforts with progress tracking at each stage. Using unique identifiers encoded in QR codes and a taxonomic database, a research team can sample herbarium specimens, efficiently record the sampling event, and capture specimen images. After sampling in herbaria, images are uploaded to a citizen science platform for metadata generation, and tissue samples are moved through a simple, high‐throughput, plate‐based herbarium DNA extraction and sequencing protocol.

**Results:**

We applied this sampling‐to‐sequencing workflow to ~15,000 species, producing for the first time a data set with ~50% taxonomic representation of the “nitrogen‐fixing clade” of angiosperms.

**Discussion:**

The approach we present is appropriate at any taxonomic scale and is extensible to other collection types. The widespread use of large‐scale sampling strategies repositions herbaria as accessible but largely untapped resources for broad taxonomic sampling with thousands of species.

Herbaria are critical resources for documenting plant diversity, and collectively the global network of 3100 herbaria, with 390 million specimens (Thiers, 2021), encompasses foundational information on the geographic, temporal, and taxonomic variation of plants (Heberling and Isaac, 2017; Soltis, 2017; Willis et al., 2017; Heberling et al., 2019). Beyond these uses of herbaria, material taken from herbarium specimens has played an important role in molecular (DNA) systematics since the early 1990s (Taylor and Swann, 1994; Savolainen et al., 1995), and the inclusion of herbarium‐derived samples in molecular phylogenies is quite common (Savolainen et al., 1995; Telle and Thines, 2008; Staats et al., 2013; Jordon‐Thaden et al., 2020). However, DNA quality and quantity vary depending on the age and quality of the specimen, method of preservation and storage, and taxon (Neubig et al., 2014; Forrest et al., 2019). Until recently, most herbarium‐derived DNA samples were used for PCR amplification of specific loci, coupled with Sanger sequencing, an approach that requires either high‐molecular‐weight DNA or a strategy to amplify and assemble a target locus from a series of small regions. Techniques to increase the yield of specimen‐based extractions, such as rolling circle amplification (also known as multiple displacement amplification), have been applied successfully, but are most effective when DNA quality is fairly high but yield is low (Brockington et al., 2008). The need for minimally degraded DNA for successful PCR and Sanger sequencing limits the use of herbarium specimens in studies that rely on this approach, where specimens have typically been used to fill gaps in Sanger sequencing–based data sets generated mostly from field‐collected materials rather than as primary sources of DNA.

The ultimate goal of plant phylogenetics is to reconstruct the phylogeny of all of the nearly half a million species of green plants, and many studies require large phylogenetic trees with dense species sampling to address specific evolutionary and/or ecological questions. To date, the largest phylogenies for plants have been based on opportunistic sampling of data available on GenBank (e.g., Zanne et al., 2014; Smith and Brown, 2018). However, DNA sequences remain unavailable for most green plant species, and the distribution of those data is highly non‐random (Hinchliff et al., 2015; Folk et al., 2018), leading to significant limitations for phylogenetic reconstructions. First, the genetic loci that can be used in a study are limited to what has been deposited, often focusing on a small number of high‐copy plastid or rDNA loci for technical reasons related to PCR amplification and Sanger sequencing (Doyle, 1993). This limitation constrains the number of loci and nucleotides, the evolutionary properties of the loci, and ultimately the ability to resolve phylogenetic relationships, especially because the number of characters (nucleotides) available to resolve phylogenetic placements relative to the number of phylogenetic tips yields trees with high uncertainty (Simmons and Goloboff, 2014).

In addition to limited locus coverage, incomplete and poorly vetted data create two additional limitations. First, although molecular phylogeny reconstruction is surprisingly robust to missing character data (Wiens, 1998, 2003; Wiens and Morrill, 2011; Jiang et al., 2014), the introduction of non‐random missing data (Simmons, 2012a, b) can have unpredictable consequences. GenBank contains strong biases across geographic regions, regional socioeconomic status, and phylogenetic dimensions (including clades containing economically important and charismatic species), as well as numerous other factors (Meyer et al., 2015; Folk et al., 2018). Second, use of public data largely requires users to trust the identification of the species attached to the sequence. While, in principle, GenBank data should be associated with specimen vouchers, voucher information is often missing, and metadata standards for voucher specimens are applied inconsistently (Funk et al., 2018; Tahsin et al., 2018; Troudet et al., 2018). Thus, access to voucher data for a large data set cannot generally be obtained without extensive labor and specialized methods (Pelletier and Carstens, 2018). Limited access to digitized voucher specimen information, including images and metadata such as localities, not only prevents researchers from verifying the taxonomic identity or geographic precision of a voucher specimen (and the DNA sequences derived from it), it also prevents a researcher from acquiring morphological or other character information for the same specimen from which the DNA sequences were obtained. In sum, any phylogenetic hypothesis generated from a limited set of loci available online is likely to provide a highly incomplete account of evolutionary history.

Fewer than one third of named green plant species have usable DNA sequence data in GenBank and are represented in large‐scale phylogenies (Hinchliff and Smith, 2014; Hinchliff et al., 2015). This limitation will remain without new approaches that can simultaneously overcome technical challenges and reduce the labor required to rapidly obtain sequence data for large numbers of species for phylogenetic inference. Whereas sequencing methods have rapidly improved, sample acquisition for molecular studies has not seen a radical transformation and remains among the greatest bottlenecks for large phylogenetic projects. Herbaria, as vast repositories of genetic material, provide huge reservoirs of species diversity to meet this need. Sampling tissue from herbarium specimens is far cheaper and more efficient than efforts to obtain silica‐dried or fresh material in the field. Furthermore, some species may be known from only one or a few localities and may be difficult or impossible to recollect, but are readily obtained from herbarium collections. Moreover, the use of DNA from known specimen vouchers means that DNA sequences from an individual specimen may be combined with trait and locality data obtained from the very same specimen.

Next‐generation sequencing approaches are a natural match with herbarium materials and have greatly lowered barriers to obtaining sequences from museum specimens, which typically yield highly fragmented DNA. Many next‐generation sequencing technologies, such as Illumina, inherently require short fragments of DNA, which are sequenced and then assembled to build longer contiguous sequences. Among the many approaches that have been used to sequence herbarium specimens, Hyb‐Seq (one of several terms referring to targeted capture of specified loci through hybridization of probes to genomic DNA, including organellar loci) is a popular and accessible application that uses short‐read sequencing to generate phylogenetic data sets (Stull et al., 2013; Mandel et al., 2014; Weitemier et al., 2014; Dodsworth et al., 2019; Johnson et al., 2019) and is frequently used for herbarium specimens given its often high performance on very degraded DNAs (Weitemier et al., 2014; Folk et al., 2015; Forrest et al., 2019; but see Straub et al., 2012; Beck and Semple, 2015). Hyb‐Seq also has a key advantage in its flexibility: a single bait set can effectively target and sequence DNA across a huge phylogenetic breadth (Mandel et al., 2014; Johnson et al., 2019), but the technique is also suited to projects focusing on temporally shallow evolutionary problems (Folk et al., 2015).

Considered together, rapid sample acquisition from herbaria and high‐throughput next‐generation sequencing form a powerful yet underused strategy to generate large phylogenies. Here, we describe an accessible method to efficiently and rapidly sample herbaria to enable such large‐scale phylogenomic projects. We present a validated workflow and a data management system that we term a Specimen‐to‐Laboratory Information Management System (SLIMS), coupled with links to downstream wet lab protocols. We used this system to build a massive Hyb‐Seq data set for the “nitrogen‐fixing clade” of angiosperms, a diverse and species‐rich clade within the rosids containing more than 30,000 species in four orders: Fabales, Fagales, Rosales, and Cucurbitales. We aimed to assemble herbarium samples and generate Hyb‐Seq data for ~50% of the species across the clade and met this target in less than two years. Based on our experience, we provide a series of clear protocols and best practices to inspire future efforts at unlocking the biodiversity vaults that are our herbaria. We present our work as a case study in how to manage a large phylogenomics project and provide a set of software tools and protocols for each of the components. Because many of the software design decisions we made were customized for our research project, instead of a consolidated software package, we provide the code base as a set of open‐source modular scripts with documentation of each function so that parts of our approach can be included in the applications of other researchers using other management systems and protocols.

## METHODS

### Herbarium sampling

#### Sampling workflow design

**FIGURE 1:**
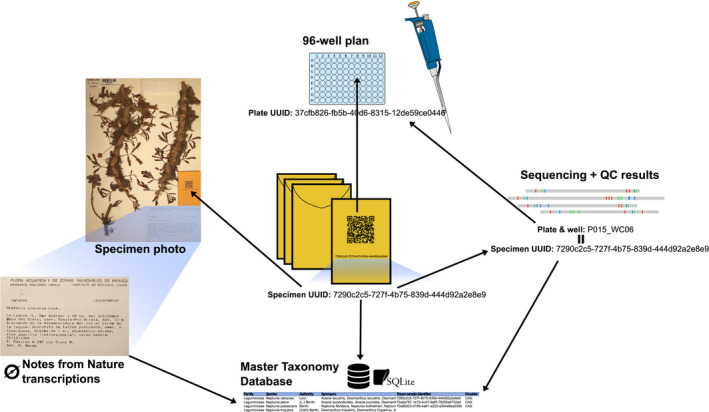
Visual representation of the workflow. Sample envelopes with QR code are placed in the center of the image to represent their centrality in tracking steps of the workflow. Arrows indicate relations to specimen images and transcription efforts, wet lab and sequencing efforts, and finally arrows repatriating results to the SLIMS database (bottom).

We greatly increased the efficiency of herbarium sampling by avoiding two key bottlenecks of traditional destructive sampling workflows: (1) time spent populating a manually entered spreadsheet with several minimal data fields during a sampling event, and (2) time spent duplicating this information by labeling collection envelopes. Our approach essentially comprises: identifying herbarium sampling events with pre‐generated universally unique IDs (UUIDs); populating the UUID and the institutional specimen barcode into a pre‐made, cloud‐based taxonomic spreadsheet; and rapidly capturing new specimen photos containing a pre‐printed sampling envelope with the UUID encoded as a QR code (Fig. 1). UUIDs are used as the central identifier to associate specimen collection events with specimen metadata and downstream products such as DNA sequences; UUID links for biodiversity data are reviewed by Nelson et al. (2018).

We generated an internal taxonomic resource by subsetting a complete record of *The Plant List* (version 1.1; http://www.theplantlist.org) to the nitrogen‐fixing clade, using SQLite queries in R version 4.0.3 (R Core Team, 2020). The fields we maintain include the following from *The Plant List*: family, scientific name, synonyms (important for resolving taxonomic disagreement), and a taxon identifier field. In addition, we added one project‐specific field (project UUID) and two fields that link to herbarium providers (provider Index Herbariorum acronym [Thiers, 2021] and specimen provider barcode) for all accepted names. The resource was instantiated as a Google Sheet and served a key role when working in herbaria. When a herbarium specimen was sampled, we recorded a unique identifier associated with that sample (see below for details) based on the specimen identification. This provided a key link between samples and valid species names, which is a core relationship we track for all downstream steps. This approach also assures that we maintain one master taxonomy usable for connecting multiple data products generated in this work. We note the innate value of an interactive cloud‐based spreadsheet for providing a means for simultaneous sampling, obviating the risk of duplicated effort. As a pragmatic example, we often had two or three separate teams working simultaneously on sampling to speed efforts.

We never manually entered a unique identifier into spreadsheets. Rather, we developed a low‐cost system where UUIDs were pre‐generated (script https://github.com/rafelafrance/nitfix/blob/master/nitfix/print_uuids.py), printed on sampling envelopes (see Appendix 1), and could be read instantly with barcode readers to associate them with a collection event. Specifically, we pre‐printed UUIDs encoded as QR codes on the front of coin envelopes into which each tissue sample was placed. This approach replaces either handwritten or machine‐printed labels populated with information at the time of sampling and speeds up the collection process while reducing record‐keeping errors. Pre‐assigning labels to envelopes also prevents identifier duplication across simultaneous collaborative sampling efforts. The UUID is a key identifier maintained throughout the project to associate photographs of specimens, tissue sampling events (including taxonomy), sequencing outcomes, and other data products. UUIDs were generated using Python’s UUID library. We used the program TAGGIT Pro version 8.50 (SATO America, Charlotte, North Carolina, USA) to generate 15,000 QR codes for the UUIDs and printed these labels on #1 coin envelopes by feeding individual envelopes into a Brother HL‐4150CDN printer (Brother International, Bridgewater Township, New Jersey, USA) using the front feeding tray.

The full recommended workflow for herbarium sampling is outlined in Appendix 1, including brand names and part numbers for equipment. Briefly, a collecting team (1) chooses a specimen and (2) scans a QR‐encoded envelope to populate the UUID field, which is always associated with a valid taxon name. We also scanned in the provider’s specimen barcode, if available. Next, (3) the destructive sampling is performed, (4) a pre‐printed destructive sampling slip is affixed, and (5) a photograph of the specimen is taken including the envelope with its QR code face‐up, in a position not obstructing annotation slips or label data. Appendix 1 contains a full set of guidelines based on our experience with protocols that have worked in many of the world’s largest herbaria, and we give a full set of caveats and best practices across different curatorial practices relevant to destructive sampling. Prior to extraction, samples were stored in coin envelopes kept in the dark within sealed Ziploc bags at room temperature. Some samples were stored up to two years before processing. Given the museum storage of these specimens in similar conditions up to more than 100 years, we expect minimal success variance due to different processing times.

### Wet lab processing

Here we provide details of our wet lab procedure from DNA extraction and sample management to sequencing submission. We note that while in our case this was tightly paired with the SLIMS system, primarily at the level of extraction plate layouts, sample submission decisions, and QC statistic management, many aspects of the protocol are portable and compatible with other specimen and lab management solutions.

#### 96‐well plate DNA extraction

We performed high‐throughput DNA extractions in a 96‐sample plate format that enabled the processing of 400–600 samples per person‐week. Key to scaling up a traditional plant phylogenetics wet lab procedure was the use of a low‐cost extraction management system, in this case using a cloud‐based Google spreadsheet that we developed. This spreadsheet was customized using a JavaScript (Google Apps) script attached to Google Sheets. The spreadsheet includes a new menu item that allows single‐click creation of a new 96‐well extraction plate template. Each new plate template could be added sequentially to the spreadsheet, allowing a manager to have a single sheet for all plate extractions. Each plate template comprised a 96‐well plate layout and fields for metadata, including a plate UUID corresponding to a pre‐printed label on the extraction plate (plate UUID label stickers were printed as described above for envelopes). We entered sample UUIDs by scanning the QR code on each sample packet into one of the 96 cells in the plate template, each corresponding to a plate well. Finally, we loaded each herbarium tissue sample from a QR‐coded envelope into the corresponding well for movement to the DNA extraction protocol below.

The plate template was critical for creating and enforcing regular structures for data entry, an important consideration for collaborative projects where it is important to avoid unnecessary labor from idiosyncratic data practices. The template script also sets up fail‐safes to streamline and audit the data as information is being entered into worksheets. In particular, the script disallows certain entries, such as duplicated sample IDs, in order to detect errors before they are ingested into the database. The end result is a single Google Sheet to manage all the extractions performed and facilitate ingestion into the main database as described below. This consolidated extraction datasheet helps simplify tracking, benefitting from version control built into Google Sheets.

The DNA extraction protocol we developed is described in detail in a protocol in Appendix 2; we briefly summarize it here. Extraction plates were set up using the Genesee Scientific (Rochester, New York, USA) 1.2‐mL mini tube system (catalog number 14‐363) with two 4‐mm stainless steel beads per tube. We ground 20–30 mg of herbarium tissue into a fine powder using a MiniG Automated Tissue Homogenizer (SPEX SamplePrep, Metuchen, New Jersey, USA) at 1500 rpm for 60–120 s at room temperature. Thorough homogenization of dry herbarium specimen tissue is critical for adequate penetration of extraction reagents to enable isolation of sufficient quantities of DNA for next‐generation sequencing; we have found the choice of homogenizer and tube compatibility are critical elements. After grinding, 500 µL of 2× cetyltrimethylammonium bromide (CTAB) buffer was added to each tube using a Rainin E4 12‐channel pipette (Mettler Toledo, Columbus, Ohio, USA). Samples were homogenized at 900 rpm for 20 s and incubated at 65°C for 60 min in an incubation oven. After incubation, the lysate was transferred to a new set of tubes to reduce the total volume and allow for the addition of chloroform without tube overflow. DNA was isolated and purified twice by adding an equal volume of 24 : 1 chloroform : isoamyl alcohol and transferring the supernatant to a new set of tubes. DNA was precipitated by an 8–24 h incubation at −20°C with 0.08 volume of cold 7.5 M ammonium acetate and 0.54 volume of cold isopropanol. DNA pellets were washed two times with 500 µL of cold 70% ethanol, and dried DNA pellets were resuspended in 33 µL of molecular‐grade water. Extracts were transferred immediately to 96‐well microplates using an LTS 12‐channel pipette (Mettler‐Toledo Rainin LLC, Oakland, California, USA), and plates were sealed with AlumaSeal foil (Excel Scientific, Victorville, California, USA).

#### Sequencing submission, processing, and metadata ingestion

Samples were briefly stored at −20°C and submitted to RAPiD Genomics (Gainesville, Florida, USA) for quantification, library preparation, targeted enrichment using a custom biotinylated RNA bait set, and multiplex sequencing using the Illumina HiSeq. Standard Illumina library processing was performed by RAPiD Genomics with two modifications: (1) no DNA shearing was performed, and (2) bead‐based cleanup was performed after DNA normalization and prior to library building. Our capture kit was partly standard in design, targeting exonic regions of 100 conserved low‐copy markers for phylogenetic studies, as developed using the MarkerMiner pipeline (Chamala et al., 2015) with a set of rosid transcriptomes both within and outside the nitrogen‐fixing clade, as derived from the 1KP project (Matasci et al., 2014). However, we also included a set of 129 functional loci related to nitrogen‐fixing symbioses to investigate the gain and loss of symbiotic competency in species of the nitrogen‐fixing clade. In total, the bait set comprises approximately 34,000 probes targeting a capture space approximately 377,121 bp long (116,680 bp for phylogenetic loci; 260,441 bp for functional loci). The details of the loci and kit will be published in a future work; results presented here pertain to the phylogenetic loci.

We developed a simple script to convert the Google Sheet plate information into a format compliant with RAPiD submission metadata requirements. Samples on extraction plates were quantified via PicoGreen (Quant‐iT PicoGreen dsDNA Assay; Thermo Fisher Scientific, Waltham, Massachusetts, USA) and robotically reformatted at the sequencing provider to exclude samples with total DNA below 10 ng (approximately 5% of project samples), because initial tests indicated that total quantities of DNA as low as 10 ng were suitable inputs for genomic library preparation. During reformatting we also excluded samples from genera for which we had met sequencing goals as determined by progress reports (see below).

### Data management and analysis

#### SLIMS database design and deployment

We implemented a lightweight SLIMS, summarized in Fig. 1, for handling project data in a unified way commensurate with the throughput needed for a large phylogenomics effort. The SLIMS consists of three major components. First, the central component is an SQLite3 database storing most project data. The general design is to have Python scripts download and ingest all of the raw data, typically in the form of Google Sheets, into the database as close as possible to the original format. Inputs are then processed, error‐corrected, and merged into a usable format. To prevent chaotic data outputs, we have a few key values that are tracked through the system and for which we enforce data integrity. The primary key field is the sample UUID, described above. It is used to track data from sample collection and initial imaging, to DNA extraction, through DNA sequencing. Other important key fields are the taxonomic name and any external IDs assigned by other organizations.

Aside from the tables that contain the raw data ingests, there are some core internal tables in the database. There are two tables for ingesting the images, one that links the image file to the scanned sample ID (QR code) and one that contains all of the errors that occur during scanning, such as unreadable or (extremely rarely) duplicated QR codes. There is also another primary set of tables for the taxonomy—one containing taxonomy data, and another that links taxa to one or more sample IDs—and finally a table to hold any unresolved sample ID errors such as sample IDs mapping to multiple taxa. Another table holds the data related to the sample extractions, keeping track of sample assignments to extraction wells and to reformatted sequencing wells. The final pair of tables is used to track the samples through the sequencing process.

The second central SLIMS component comprises a set of Python scripts that perform data processing. Most of the scripts form the backbone of the ingestion and reporting process. Because there are dozens of scripts for each of these processes, we have a makefile that simplifies the process. An end user can run the entire process end‐to‐end via a “make all” command, or a set of scripts can be run for a particular function like scanning images for QR codes or ingestion and auditing of the taxonomic data.

**FIGURE 2:**
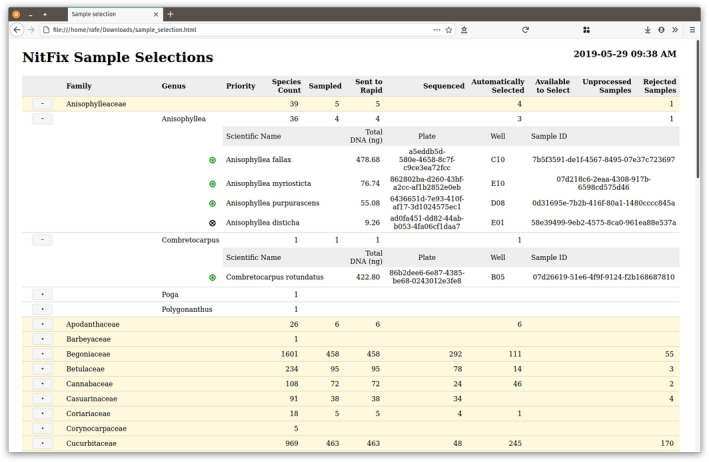
A screenshot of a sample selection report. The report is used to prioritize which samples to select for sequencing. There are three levels (family, genus, species) of reporting. The family and genus levels are collapsible by clicking on the buttons (“+” or “‐”) to the left. The meanings of the glyphs are given when the mouse hovers over them. Green marks indicate that the sample met the criteria for sequencing, and the black cross indicates that the sample was rejected for sampling because the yield was too low. See an interactive example at: https://rafelafrance.github.io/nitfix/assets/sample_selection.html.

The final SLIMS component is the reporting system (Fig. 2). Here we create standalone interactive browsable HTML reports and accompanying CSV files for downstream analysis based on needs from end users (example at https://rafelafrance.github.io/nitfix/assets/sample_selection.html). The HTML reports are single‐file reports that can be distributed to scientists to run locally without using an HTML server. These reports are not simply static HTML but have functions built in that can be used to search, filter, and otherwise examine the data. These reports were critical for determining sequencing prioritizations and coverage quality. Reports also provide a means to track sequencing success rates and the progress of samples through the steps of the workflow. Reports were generated by request after their format was determined, based on team input, to display the most salient data for decision‐making. Ultimately, among the products of our work on the nitrogen‐fixing clade will be a forthcoming web interface for serving DNA sequences and other data products from the project along with associated metadata extending the HTML reports developed here.

### Specimen metadata generation

#### Specimen transcription

Transcribing voucher metadata is a substantial bottleneck that can be avoided during the herbarium sampling process. We designed an approach for use during sampling visits to herbaria that is limited to (1) obtaining physical samples, (2) associating these to identifiers, and (3) capturing specimen photographs. We subsequently transcribe labels from those photographs to associate specimen metadata with the related collection events.

Given the scope of our project, we used a citizen science approach, uploading our voucher photographs for label transcription to the Notes from Nature platform (Hill et al., 2012), which is itself part of the Zooniverse (https://www.zooniverse.org/) roster of projects. Notes from Nature is organized around “expeditions,” which are typically thematic sets of imaged specimens of a manageable number. Years of optimizations have suggested that image sets of 2000–3000 are large enough to avoid excessive set‐up overhead, but small enough for public participants to be motivated by a reasonably sized completion goal. Setting up a Notes from Nature expedition is a straightforward process; images and a “manifest” file (containing image and sample identifiers and other associated metadata) are uploaded via a Zooniverse toolset called the Project Builder. The Project Builder also provides a set of menus for determining what is targeted for transcription. While it is now feasible for providers to do most project set‐up on their own, Notes from Nature and Zooniverse staff help oversee the process and give final approvals for launches. Providers also need to develop clear help and introductions to expeditions and spend time directly interacting with volunteers to handle digitization problems as they arise.

Transcription “expeditions” for specimens of the nitrogen‐fixing clade were initially launched with the goal of assembling complete label transcription, but this process was very slow, given a relatively high percentage of labels in non‐English languages (approximately 22.5% overall, with higher percentages in some collections) and the often complex locality descriptions on the labels. In order to strategically capture only those key fields needed for a large phylogenomic investigation, we scaled back requests for whole label transcription, and instead asked public participants to provide only minimal data on administrative units (such as county, state, and country), date collected, collector, and collector number. For administrative units, we relied on drop‐down lists, both to obviate the need to type unit names and to enforce standard input. To date, all the minimal data transcriptions for our project have been completed, and subsequent efforts to obtain detailed locality descriptions are underway.

Here again, UUIDs were central to linking transcriptions to the centralized SLIMS database. Each photograph had an envelope with a QR code associated with it, which was automatically harvested using a script we developed to segment the QR code from the specimen image and read its contents. This provided a simple way to ingest the photographs into the main database using the UUID as a primary key without manual entry. It also assured that image and sample metadata could remain associated and passed along during transcription workflows.

## RESULTS AND DISCUSSION

### Throughput

For herbarium sampling, the average effort was 0.16 ± 0.04 person‐hour per specimen excluding travel (i.e., 9.5 ± 2.6 person‐minutes). We worked in teams of two to five per herbarium sampling trip, averaging 179 ± 52 specimens per day (maximum 400). For DNA extraction, after a learning curve of approximately one week, our average throughput was 0.09 person‐hour per specimen (i.e., 5.3 person‐minutes; approximately 450 samples per week). We typically worked in groups of one or two for wet lab work. Our final collection effort included 16,562 species in 1259 genera from 11 herbaria (BRIT, CAS, F, FLAS, HUH, KUN, MO, NY, OS, TEX, US [Thiers, 2021]). Of these, 14,492 samples were moved forward to sequencing.

### Error rates

Our error rate for sampling was approximately 1.21%, including unreadable QR codes (0.28%) and 76 pairs of images with duplicated barcodes due to printer errors (0.95%); note that these percentages do not add up due to overlapping errors. Our focus was on error detection and management; we generally did not attempt to fix the tissue samples generating these errors because they affected relatively low‐priority taxa that did not need to be moved to sequencing. However, most of these errors were manually resolvable and could be disambiguated by closely investigating the images and associating them with specific collecting trips.

### Patterns of locus recovery

**FIGURE 3:**
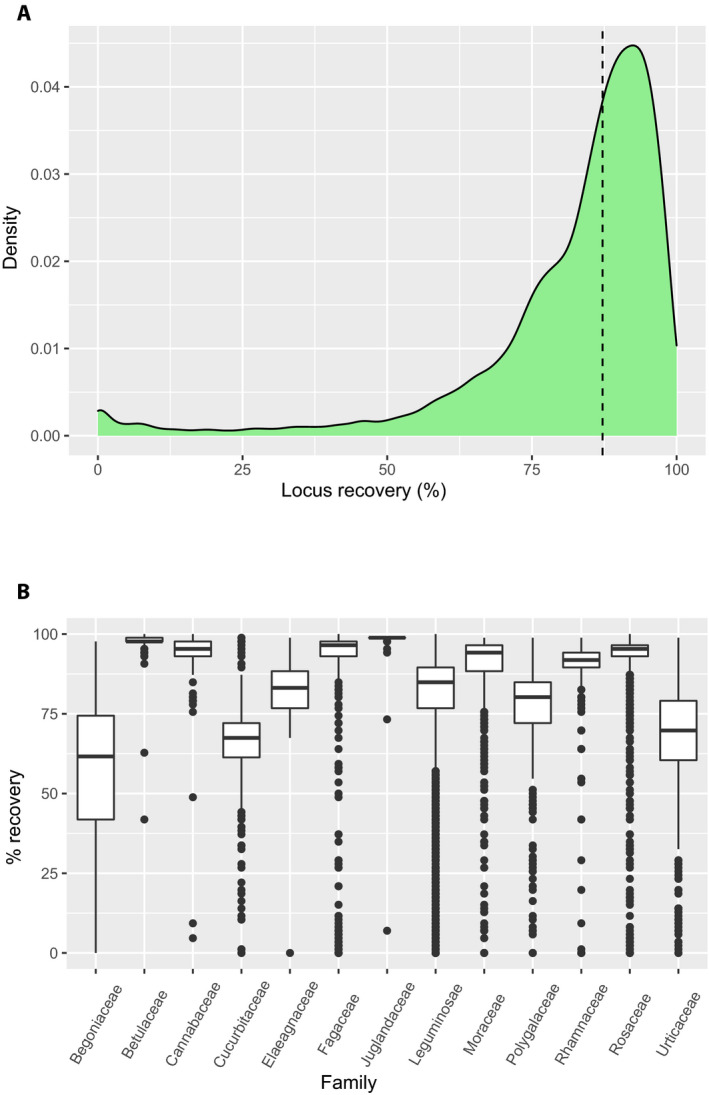
Locus recovery statistics. (A) Kernel density plot of overall locus recovery; the dotted vertical line is the median (87.2%). (B) Boxplots of locus recovery for every family with at least 50 samples.

There was a 0.20% sequencing failure rate (failure defined as less than 5% of loci assembled); on average, 87.2% of loci were assembled per sample (Fig. 3A). The number of targeted loci successfully assembled (here, success was defined as at least 6× reference coverage and 350 bp assembled, excluding 14 high‐copy loci) was significantly associated with taxonomic family (one‐way ANOVA, *P* = 2e‐16), although the effect size was relatively weak (*η* ^2^ = 0.1589, that is, about 16% of the variance explained by family). Families associated with lower recovery (Fig. 3B) include those well known for being rich in secondary compounds (e.g., Begoniaceae, Leguminosae, Urticaceae). We also tested for an effect of distance from the baits, using a recent seed plant tree (Smith and Brown, 2018), successfully mapping 78% of taxa to calculate patristic distance between the sample and the baits. The calculations were implemented in Dendropy (Sukumaran and Holder, 2010), taking the distance to the nearest bait design taxon (i.e., *Glycine soja* Siebold &. Zucc., *Juglans nigra* L., *Polygala lutea* L., *Quercus shumardii* Buckley, *Quillaja saponaria* Molina, *Rosa palustris* Marshall, or *Ulmus alata* Michx.). Although there was a significant relationship between the evolutionary distance to the baits and the number of loci assembled (*P* < 2.2e‐16), the effect size was very low (adjusted *R* ^2^ = 0.016), suggesting that evolutionary distance explains only a trivial amount of the lack of capture success we observed (see also Johnson et al., 2019). Further assessment of sample age, quality, and other factors is warranted but beyond the scope of this contribution.

### Dynamic SLIMS reporting

Critical to a project of this size and scope is managing project data transparently and flexibly and capturing errors as they arise. The novelty of our approach lies in linking herbarium sampling events with wet lab information management in a single integrated database and reporting system. As opposed to traditional project management, which is often founded on idiosyncratic data formats and one‐off data integrations, we use integrated data tables to easily perform standard data reporting tasks in a way that is responsive to collaborators. UUID tracking streamlines standard tasks that would normally be made difficult by separate data structures, such as linking original specimens (captured by photos and institutional barcodes) with DNA quality data (delivered to us as summary spreadsheets) and minimal specimen metadata (delivered from completed Notes from Nature expeditions). As well as easily performing custom queries, generating as‐needed sampling reports allows us to track project progress and collaboratively discuss adjustments to sampling and wet lab efforts.

### Crowdsourcing transcription

Collecting trips, even to herbaria, are expensive and labor‐intensive; therefore, another critical element to overcoming the herbarium sampling bottleneck was to deliberately disconnect the act of sampling from capturing minimal data. In the largest collections, most specimens remain undigitized, motivating us to capture these needed data via specimen imaging and citizen science efforts. We focused on completing the minimal data fields that are most relevant to a large phylogenomic project, enforcing data entry standards to ensure high‐quality transcriptions. We also note that while triplicate scoring is often important for fields with difficult‐to‐transcribe content such as locality descriptions, in our expedition design we relaxed this requirement for the simpler fields that could be entered using a controlled vocabulary and had an associated drop‐down user interface, such as date collected and higher administrative units (e.g., country and state).

An additional benefit of our use of the Notes from Nature platform is the ability to return our data to specimen providers and contribute to improvement of the collections we used for our study. Depending on the collection we worked with, we were in some cases able to mount joint transcribing expeditions to fully digitize specimens. For other collections not already set up for crowd‐sourced transcriptions, we returned partial metadata fields that curators could follow up with additional expeditions to complete the specimen records. Crowdsourcing specimen transcription also allowed us to easily prepare destructive sampling manifests to send to curators.

### Comparison with traditional workflows

Many plant phylogeneticists will be familiar with a traditional destructive sampling workflow in herbaria, in which metadata on sample provenance are recorded by hand in a spreadsheet during sampling. Even if minimal metadata are captured (typically a determined name, collector, and collector number), these are time‐consuming to record and often handwritten in duplicate on sample labels, a substantial time and money investment for large projects. We have avoided these steps with some simple modifications. Assigning a UUID during the sampling event obviates the need to record specimen provenances at the point of sampling as long as these are associated with the UUID. Capturing a specimen image containing a scannable UUID means that specimen metadata can be captured separately and that these data can be associated with UUIDs and photographs automatically. Sometimes it is necessary to record notes on specimens; while we primarily used these only for early pilot sampling events, we were able to increase throughput in notetaking by pre‐encoding common specimen notes into pre‐printed QR codes to rapidly enter typical data (e.g., specimen color, herbarium source).

The benefits of our approach are not limited to the herbarium. Experience demonstrates that large projects are sometimes driven by chaotic spreadsheet trading via email or file‐sharing services. We sought to prevent irregular data‐handling practices at the outset of our investigation to avoid not only errors but the potential for large amounts of needlessly expended effort. As an example, rather than laboriously linking spreadsheets by hand, querying a relationship between sequencing success and specimen age requires a simple SQLite query to generate a CSV with selected headers, which can then be imported in a straightforward way into statistical software for investigation. Interactive HTML reporting was instrumental in diagnosing early sampling efforts and pilot sequencing runs at the beginning of our project to assess progress and success. Sample reports will also eventually form part of our data dissemination practices and facilitate broad reuse.

### Workflow components

We have presented a complete herbarium‐to‐sequencing workflow that was developed based on our own project needs, including such issues as sampling priorities and QC statistic reporting. It is unlikely that we could anticipate all future project needs or easily allow major changes for different types of projects, such as those focusing primarily below the species level. With that in mind, rather than trying to present a unified and generalizable software solution, we have focused on providing a series of modular scripts with operation instructions (https://github.com/rafelafrance/nitfix/tree/v0.1.1‐SLIMS) for tasks from UUID generation to sample plate formatting in Google Sheets. It is not necessary for other large‐scale phylogenomics projects to use all portions of the approach we developed for the nitrogen‐fixing clade of angiosperms. Future users of our work may wish to use only parts of the sampling and wet lab protocol we present, and this modular script design makes it straightforward to reuse relevant portions of the SLIMS code base for other projects.

### Outlook for large‐scale phylogenetics

The scale of phylogenomic investigations is increasing, with projects that include hundreds of samples becoming increasingly common (Ruhfel et al., 2014; Leebens‐Mack et al., 2019; Zhu et al., 2019), but rapid workflows keeping pace with an increasing interest in large phylogenomic data sets have been lacking. Anecdotally, many investigators are finding that next‐generation sequencing methods have lowered barriers to generating large sequencing data sets so much that sample acquisition and data analysis now tend to be the pre‐eminent bottlenecks. Herbaria, as critical resources documenting plant diversity, have been central to enabling large‐scale investigations (Soltis, 2017; Soltis et al., 2018). Considerable work has been dedicated to high‐throughput digitization workflows in herbaria (e.g., Nelson et al., 2015); parallel methods to enable other downstream analyses of herbarium specimens may one day enable much of today’s collections to be associated with molecular and other data reliant on destructive sampling. We anticipate that high‐throughput sampling approaches like that presented here will be a standard part of the phylogenomics toolkit in future large‐scale projects.

## AUTHOR CONTRIBUTIONS

R.A.F., R.P.G., and H.R.K. conceived the work with contributions from all authors; R.A.F. prepared the first draft with contributions from H.R.K., R.L., and R.P.G.; all authors substantially contributed to the final manuscript.

## Data Availability

Code for the SLIMS system presented here is maintained on GitHub at https://github.com/rafelafrance/nitfix, with a release representing the version presented here at: https://github.com/rafelafrance/nitfix/tree/v0.1.1‐SLIMS. This repository includes modular functions for the tasks described here as well as step‐by‐step documentation.

## APPENDIX 1. Herbarium sampling protocol.

### Part I. Create the sampling spreadsheet and put together your sampling kit

1. Prior to any sampling, create a Google Sheet, which we refer to hereafter as the “Taxonomy‐Sample UUID” datasheet, containing columns for the following: (a) currently valid names, which should be populated before any herbarium sampling begins; (b) known synonyms (as a comma‐ or semicolons‐delimited list), also already filled in prior to sampling; (c) an assigned project sampling identifier, to be empty upon starting the project; (d) the herbarium acronym and the herbarium identifier (that is, any already existing identifiers on the specimen, which we filled in only if it could be easily scanned from a barcode), which also are empty upon start of the project; and optionally (e) extra columns for family names and notes. This spreadsheet is a crucial part of protocol and effectively links a physical sample to a sample identifier and a taxon name.
2. We created a kit that traveled with us and was used during sampling. That kit included the following items: (a) multiple high‐quality digital cameras with the number dependent on the number of teams you have working; we recommend a camera brand that is known to be suited for indoor, fluorescent, low‐light conditions; (b) a large number of coin envelopes (#0 or #1) with a pre‐printed QR code on the front of the envelope; that QR code contains a UUID used as a sample identifier; (c) a handheld barcode scanner used to read the QR code as well as other barcodes on samples; (d) an articulating arm camera mount that can be clamped to a table; (e) multiple pairs of forceps; (f) printed labels on archival paper to be affixed to herbarium sheets denoting destructive sampling; (g) archival glue for herbarium slips and glue sticks for sample envelopes; (h) spare camera batteries, especially important as camera battery life was often limiting.
3. Although there are many reasonable choices and digital camera models are constantly being updated, we note these key options that proved to be worth the cost for the following sampling kit items (all other items can be standard off‐the‐shelf): (a) *digital camera:* Ricoh GR II (Ricoh Company, Tokyo, Japan), which is highly performant in ambient fluorescent lighting; (b) *table‐clamping collapsible camera mount:* Manfrotto 035RL Super Clamp and Manfrotto 196B‐2 143BKT 2‐Section Single Articulated Arm (Manfrotto, Cassola, Italy); (c) *barcode scanner:* Kercan KR‐230‐EIO (Shenzhen, Guangdong Province, China); the model should be able to capture serial barcodes and QR codes to enable capture of any previous identifiers; barcode scanners work much like USB keyboards and must be checked for operating system compatibility; normally they are programmable and should be set to follow the scan with a carriage return; finally, avoid models needing an independent power supply.

### Part II. Workspace setup

1. Set up a workstation with a laptop, barcode scanner, and table‐mounted digital camera, and open the master taxonomy sampling datasheet (loaded as a collaborative Google Sheets spreadsheet). Multiple collectors can work in tandem as the shared taxonomy spreadsheet prevents redundant entries.
2. Position the camera above a clean desk space at a height and angle such that a herbarium sheet positioned on the table below fills the frame. This position can be marked with tape or pencil for quick placement.

### Part III. Sample evaluation

1. Locate the most recent species name determination in the Google Sheet via search functions, being sure to include matches in the synonym field. Rarely, homonyms must be resolved via the use of taxon authorities; use the authority field for accepted names, and as necessary perform manual resolution with taxonomic resources such as http://www.theplantlist.org/.
  - Determine the most recent identification of the specimen, taking into account prevailing curatorial practices at the herbarium. Generally, this will be noted on the most recent annotation slip. Space allowing, annotation slips are traditionally placed in a sequence upwards and then leftwards of the label. Anonymous or penciled‐in IDs are found on older specimens and count as annotations; use any available dates and spatial positioning to infer the most recent annotation. Also be aware of differences between filed and annotated species, as in some collections it was traditionally common to re‐identify species only by refiling without permanent annotation. Training of samplers must stress the correct interpretation of annotation labels, as this is the most common error source for new samplers.
  - Although it may be tempting to sample in order of project priority, it is critical to follow the herbarium filing order to reduce substantial searching labor in large collections, which are often not filed according to modern phylogenetic taxonomy. When reviewing specimens, it is also important to maintain filing order between and within folders when searching, and not to unfile specimens if at all possible, as this adds labor often far exceeding the labor required to sample from and database specimens. Collections have highly diverse regional filing systems, sometimes including county‐level ordering within folders, and these are best undisturbed during the sampling process to maintain efficiency. A minority of collections require that specimens are unfiled as part of their standard destructive sampling workflows, so samplers should be prepared to make advance arrangements and warn curators of any special project needs.
2. Decide on a specimen to sample, giving precedence to curatorial guidance. In general, specimens should be selected in the following priority order:
  - Leaf color should be as green as possible, and floral color as bright as possible (if applicable, considering the taxon and developmental stage).
  - There should be substantial material on the specimen, with duplication of essential structures for identification and scientific study.
  - Consider the nature of the identification when several suitable specimens are available. Determinations by taxon experts are of high value, and specimens with full sets of features are more likely to be well‐identified than sterile or fragmentary specimens. High‐quality determinations using older taxonomies are often easier to resolve than new determinations that use up‐to‐date taxonomy but are incorrect.
  - Age can be considered in specimen sampling, and we generally avoided older samples (>100 years old) because these often lack high‐quality collection metadata, but a well‐pressed old specimen is always preferable to a new specimen with signs of specimen treatments problematic for DNA integrity. From least to most problematic, we have considered: dryer burn, fungal attack, use of the Schweinfurth method (ethanol dousing), and mercuric chloride treatment.
  - In ambiguous cases, consult with curators, clearly indicating the correct specimen filing position if a specimen must be unfiled and set aside.

### Part IV. Sampling

1. In the open “Taxonomy‐Sample UUID” Google Sheet, scan the QR code from an empty envelope to populate a UUID into the tissue identifier field for that taxon.
2. Using clean forceps, remove tissue from the voucher in accordance with destructive sampling guidelines and place it in the scanned envelope. As a brief overview of practices standard at international herbaria: (a) start with the sampling packet, but do not take detritus or material obviously not matching the main specimen, and sample from the specimen if needed; (b) prefer leaves, and prioritize obscured parts of the specimen, considering whether removal of overlapping parts may improve the visibility of diagnostic structures; (c) do not take structures typically important for identification unless they are very numerous (e.g., flowers, bracts, stipules) and avoid taking the last example of a particular developmental stage or leaf face.
3. To photograph the voucher, position the envelope on top of the herbarium sheet; it should be placed face up to display the QR code and care should be taken so that it does not obscure the label data or any annotation labels.
4. Repeat steps 6–10 until sampling targets are reached for the family or all vouchers accessioned in that herbarium have been sampled. The prefilled taxonomy sheet provides a quick visual guide to the progress toward sampling goals so collectors know how much of a particular taxonomic group to sample; for example, if UUIDs are already present in the tissue identifier field for 30 rows of a 60‐row family, that would be a rapid indication to move on to the next target family in the herbarium for a sampling target of approximately 50%; spreadsheet functions can be used to quickly generate counts for large genera.

## APPENDIX 2. NitFix CTAB DNA extraction protocol.

### Chemicals

CTAB buffer (~1 L/10 plates. Store at room temperature.)

- 100 mL of 1 M Tris (pH 8.0)

NOTE: For 1 L: 121.1 g Tris base, plus molecular‐grade water to bring to 900 mL. Then add concentrated HCl to bring pH to 8.0 (~108.5 mL HCl).

- 280 mL of 5 M NaCl

NOTE: 5 M NaCl (292.2 g/L). 1 M NaCl (58.44 g/L)

- 40 mL of 0.5 M EDTA

NOTE: For 1 L: Add 93.05 g of EDTA (i.e., disodium salt) to 800 mL of distilled water (slowly added with NaOH). Then add 10 g NAOH pellets (for pH balance).

- 20 g of CTAB for 2% CTAB (under scales) or 30 g for 3% CTAB
- Molecular‐grade water to bring to 1 L

PVP (polyvinylpyrrolidone) ~25 g/week

24 : 1 chloroform : isoamyl alcohol (~2 L/10 plates. Store at room temperature.)

- 960 mL chloroform (under hood)
- 4 mL isoamyl alcohol (under hood)

7.5 M ammonium acetate (~10 mL/10 plates. Store at 4° C.)

NOTE: 57.81 g ammonium acetate (cabinet) + 100 mL of molecular‐grade water

Isopropanol (0.5 L/10 plates. Store at 4°C.)

NOTE: 500 mL 2‐propanol

70% ethanol (~1 L/week. Store at 4°C.)

- 700 mL of 200‐proof ethanol
- 300 mL of deionized water

### Consumables

Each extraction (96 samples) uses four sets of 1.1‐mL mini tubes and 2–3 sets of caps, 2–3 foil seals, one tube rack, 6–9 layers (96) of 1000‐μL LTS tips and one layer (96) of 200‐μL LTS tips, 200 stainless steel grinding beads, 4–5 reagent reservoirs, one reusable glass reservoir (for chloroform), and a PCR plate. Details for products used are provided below.

Mini tubes and racks

NOTE: The plastic used in the mini tube systems is not made for use with chloroform, but we have found this adequate.

- Axygen Mini Tube System (Thermo Fisher Scientific, Waltham, Massachusetts, USA)

1.2 mL microtiter tubes (12‐tube strips) (Genesee Scientific, Rochester, New York, USA)

Semi‐skirted PCR plate

LTS tips

- StableStak (SS LTS 1000 μL; catalog no. 17007089; Mettler Toledo, Columbus, Ohio, USA)
- Pipette tips for Rainin LTS (1000 μL; catalog no. 24‐760RC; Genesee Scientific)
- StableStak (SS LTS 250 μL; catalog no. 17005874; Mettler Toledo)

Stainless steel grinding balls (product no. 2150; SPEX SamplePrep, Metuchen, New Jersey, USA)

### Non‐consumable equipment

Grinding ball bead dispenser

- SPEX SamplePrep 2100 Grinding Ball Dispenser (this is not the exact one we use, but the dispenser must match the ball diameter)

Geno/Grinder (MiniG Automated Tissue Homogenizer; SPEX SamplePrep)

Pipette (E4 pipette, catalog no. 17014499; Mettler Toledo)

Glass slide prep box (or something similar to serve as a reusable glass chloroform reservoir)

### Part I. Sample preparation and physical lysis with bead beating (4–8 h)

1. Arrange eight rows of 12‐strip tubes in a plate. Make sure the plate is oriented with the letters at the top and the numbers on the left side. Label both the plate and the lid with the local number and barcode. Label the left‐end tube of each row of strip tubes A–H and the right‐end tube A–H.
2. Using an acrylic bead dispenser, add two stainless steel grinding beads to two strips of tubes. Cap all tubes with strip caps.
3. For each of 96 samples, remove ~20–30 mg of dried plant tissue from the barcoded envelope with forceps and add to 1.1‐mL cluster tubes on the first rack.
  - Between samples, rinse the forceps in ethanol and then water, and dry with a Kimwipe.
  - To avoid cross‐contamination of dry airborne plant tissue, insert the forceps directly into the tube before releasing. While adding tissue to a tube, all other tubes (as many as possible) should remain capped.
  - Before you have completely finished loading the samples, perform step 4 (below). This step should be performed in advance because the extraction buffer takes about 1 h to dissolve the polyvinylpyrrolidone (PVP).
4. Make the CTAB extraction buffer.
  - Combine 60 mL of CTAB buffer and 2.4 g of PVP in a capped glass bottle and heat in an oven at 55°C or in a hot water bath to dissolve PVP. This will take ~1 h.
  - Immediately before adding the CTAB buffer to the samples, add 300 μL of ß‐mercaptoethanol and invert to dissolve.
  - These volumes are for a single plate, so increase amounts as needed.
5. Grind the plate in a Geno/Grinder at maximum speed for 2–3 min.
  NOTE: Never grind a plate with the lid on. The vise will not hold the tubes and tissue will leave the tubes, causing contamination.
  - Check that the samples are finely ground. If any sample is not ground, use a razor blade to excise the cap from the cap‐strip and use a forceps to break apart or dislodge the plant tissue as needed. Sterilize forceps between samples.
  - Replace any tube‐strips with samples that need re‐grinding and grind at maximum speed for 1 min. Repeat as needed. Leave out strips that are fully ground to avoid over‐grinding, and consolidate strips toward the center of the plate.

NOTE: You may pause after this step if it is time to go home or you need to take a break. A long pause will be needed if you have not prepared the CTAB+PVP yet. Plan ahead.

### Part II. Chemical lysis with detergent (CTAB) (20 min + 1 h incubation)

1. Add 500 μL of CTAB buffer to each sample. Preventing cross‐contamination of lysed tissue during this step is essential and requires steps a–c below:
  - Fill a reagent reservoir with ethanol for glove wash and position this to the left of the plate along with Kimwipes or paper towels. To the right of the plate, set up a full box of tips. Fill a reagent reservoir with CTAB buffer and position to the right of the tips. The physical separation on the bench of samples from open CTAB is important.
  - Carefully remove caps, one cap at a time, using each gloved fingertip in sequence to cover the open tube immediately to the left of the cap being removed. When all five fingers have been used, wash glove tips in ethanol and dry before continuing. You will need to do this once per row. Wash gloves in ethanol wash between rows. Discard caps.
  - Add CTAB buffer to open row of tubes and recap with new caps.
  - Repeat for all rows.
2. Shake in the Geno/Grinder at 900 rpm for 30 s to homogenize.
3. Cover the capped tubes with a Kimwipe and rack lid and incubate the samples in a 55°C oven for 1 h. Longer incubation time may cause too much gas to be produced, causing the tubes to open. While the plates are in the oven, rack and label a second set of tubes. Cover the open tubes with a Kimwipe and rack lid.
4. Centrifuge balanced plates for 2 min at 4000 rpm to compact tissue at base of tubes.
5. Using a multichannel pipette set to 500 μL, transfer the supernatant to a new set of tubes. Leave tubes open. It is acceptable if some plant tissue is transferred, and important to get as much buffer as possible for maximum yield.

NOTE: You may pause after this step if it is time to go home or you need to take a break.

### Part III. Phase separation with chloroform to remove proteins (1–2 h including centrifugation)

**NOTE: This is the longest and most challenging step.**

1. Aliquot 24 : 1 chloroform : isoamyl alcohol to the reusable glass reservoir. Add 500 μL 24 : 1 chloroform : isoamyl alcohol to all tubes and recap with new caps. You may reuse tips for this step if you have carefully avoided contact with the samples.
2. Shake in the Geno/Grinder at 600 rpm for 15 s to homogenize.
3. Centrifuge for 10 min at maximum speed. Carefully lift the plate out of the plate spinner to avoid disrupting layers.

NOTE: While the plates are in the centrifuge, rack and label a third set of tubes. Cover the open tubes with a Kimwipe and lid.

1. Transfer the supernatant using a manual multi‐channel pipette set to 350 μL into a barcoded rack of labeled, uncapped tubes.
2. Aliquot 24 : 1 chloroform : isoamyl alcohol to a reagent reservoir. Add 400 μL 24 : 1 chloroform : isoamyl alcohol to all tubes and recap with new caps.
3. Shake in the Geno/Grinder at 600 rpm for 20 s to homogenize.
4. Centrifuge for 7 min at maximum speed. Carefully lift the plate out of the plate spinner to avoid disrupting layers.

NOTE: While the plates are in the centrifuge, rack and label a fourth set of tubes. Cover the open tubes with a Kimwipe and lid.

1. Transfer the supernatant using a manual multi‐channel pipette set to 350 μL into a barcoded rack of labeled, uncapped tubes.

NOTE: For second supernatant transfer, use a razor blade to separate tube strips into two strips of six and load the pipette with six tips instead of 12 if desired (not necessary).

### Part IV. DNA precipitation with alcohol and salt (20 min + freezer incubation)

1. Aliquot 7.5 M ammonium acetate to a reagent reservoir. Add 20 μL 7.5 M ammonium acetate to all tubes, using a multichannel pipette set to 1200 μL on repeat mode.
2. Aliquot the isopropanol to a reagent reservoir. Add 220 μL ice cold isopropanol to all tubes, using a multichannel pipette set to 1200 μL on repeat mode.
3. Seal the plate with foil seal using a plate‐sealing roller. Invert gently.
4. Incubate in −20°C freezer overnight or for at least 5 h, whatever time is most efficient for your schedule. This long incubation time has been shown to increase precipitation of small fragments. If you are not extracting DNA from herbarium/degraded samples, this is not necessary.

### Part V. Drying the DNA pellet and washing to remove salt (1 h including centrifugations)

1. Centrifuge for 10 min at maximum speed to pellet the DNA.
2. One tube strip at a time, pour off the liquid into a waste reservoir, being careful not to lose the pellet. Tubes may be placed upside down on a paper towel to absorb extra liquid if the pellet is secure in the tube.
3. Aliquot 70% ethanol to a reagent reservoir. Add 500 μL of cold 70% ethanol to all tubes and mix in the Geno/Grinder at 800 rpm for 15 s. Cover and seal the plate with foil.
4. Centrifuge balanced plates for 5 min at maximum speed in the plate spinner.
5. One tube strip at a time, pour off the liquid, being careful not to lose the pellet.
6. Aliquot 70% ethanol to a reagent reservoir. Add 500 μL of cold 70% ethanol to all tubes and mix in the Geno/Grinder at 800 rpm for 15 s. Cover and seal the plate with foil.
7. Centrifuge balanced plates for 5 min at maximum speed in the plate spinner.
8. One tube strip at a time, pour off the liquid, being careful not to lose the pellet.
9. Leave tubes open and covered with a Kimwipe on the lab bench to dry for at least 24 h.
10. Resuspend the pellets in 33 μL of molecular‐grade water for at least 24 h prior to transferring to microplates and sending to Rapid Genomics.
